# Effect of levothyroxine supplementation in extremely low birth weight infants with transient hypothyroxinemia of prematurity

**DOI:** 10.1038/s41598-022-13927-2

**Published:** 2022-06-11

**Authors:** Shin Ae Yoon, Yun Sil Chang, Misun Yang, So Yoon Ahn, Se In Sung, Hee-seung Cho, Won Soon Park

**Affiliations:** 1 Department of Pediatrics, Chungbuk National University Hospital, Chungbuk National University School of Medicine, 1 Sunhwan-ro 776, Seowon-gu, Cheongju, 28644 South Korea; 2 Department of Pediatrics, Samsung Medical Center, Sungkyunkwan University School of Medicine, 81 Irwon-Ro, Gangnam-gu, Seoul, 06351 South Korea

**Keywords:** Endocrinology, Medical research

## Abstract

This study aimed to determine the short- and/or long-term outcomes of levothyroxine replacement therapy in extremely low birth weight (ELBW) infants with transient hypothyroxinemia of prematurity (THOP). The medical records of 335 ELBW infants with THOP were reviewed retrospectively to identify whether levothyroxine treatment affects short- and/or long-term outcomes at a corrected age of 2 years. The infants were arbitrarily grouped based on thyroxine (T4) (free T4 [fT4]) levels into group 1 (n = 142), which included infants with T4 (fT4) levels < 2.5 (0.5) ng/dl, and group 2 (n = 193), which included those with T4 (fT4) levels ranging from ≥ 2.5 (0.5) ng/dl to < 4.5 (0.9) ng/dl. Levothyroxine replacement therapy was not associated with beneficial short- or long-term outcomes in ELBW infants with THOP. Short-term outcomes, such as mortality and composite morbidities, and long-term outcomes, such as failure to achieve catch-up height at a corrected age of 2 years, were significantly higher in group 1 than in group 2, regardless of levothyroxine treatment status. Levothyroxine replacement therapy is not associated with short-or long-term advantages in ELBW infants with THOP. This study suggests that the severity of THOP may be the major determinant of adverse outcomes in ELBW infants with THOP, rather than levothyroxine treatment.

## Introduction

Transient hypothyroxinemia of prematurity (THOP) is a thyroid disorder that is characterized by transient low thyroxine (T4) levels with normal or low thyroid stimulating hormone (TSH) levels in preterm infants^1,2^. As its incidence is inversely correlated with gestational age (GA) and birth weight^3^, THOP occurs in > 50% of extremely low birth weight (ELBW) infants^4,5^. The etiologies of THOP are multifactorial and include postnatal loss of maternal placental T4 transfer and immaturity of the hypothalamus–pituitary–thyroid axis^6^. In our previous study, we observed a decrease in the incidence of THOP with increasing survival without composite morbidities in ELBW infants^7^. This finding suggests that THOP may be an epiphenomenon of non-thyroidal illness in ELBW infants.

Thyroid hormone has a critical role in the development and maturation of several tissues and organs, especially the brain^8^. THOP is known to be associated with adverse short-term outcomes, such as increased perinatal mortality and morbidities^9–16^, and impaired long-term growth and neurodevelopment^17–19^. Consequently, several studies have been conducted to determine whether levothyroxine treatment can improve the short- and/or long-term outcomes of THOP in very preterm infants^20–23^. However, the results of these previous studies are inconsistent and inconclusive^24–26^. Thus, the aim of this study was to investigate the short- and/or long-term outcomes of levothyroxine replacement therapy in ELBW infants with THOP at a corrected age (CA) of 2 years.

## Methods

### Ethics statement

The data collection procedure was approved by the Institutional Review Board of Samsung Medical Center (2021-11-137). The Institutional Review Board waived the requirement for informed consent owing to the retrospective design of this study. I confirm that all methods were performed in accordance with the relevant guidelines and regulations.

### Study population

We retrospectively reviewed the medical records of 335 ELBW infants (birth weight < 1000 g) with a gestational age (GA) between 23 and 28 weeks who were born at and admitted to the neonatal intensive care unit of Samsung Medical Center between January 2000 and July 2013 and were diagnosed with THOP based on the results of initial thyroid function tests (TFTs) performed within the first two postnatal weeks. We compared maternal and neonatal variables and short- and long-term outcomes, including growth and neurodevelopment at a CA of two years, according to levothyroxine supplementation status and the severity of THOP. The infants were then grouped according to their T4 (fT4) levels into group 1 (n = 142), which included infants with T4 (fT4) levels < 2.5 (0.5) ng/dl, and group 2 (n = 193), which included those with T4 (fT4) levels ranging from ≥ 2.5 (0.5) ng/dl to < 4.5 (0.9) ng/dl, to reduce skewing of adverse outcomes by the severity of THOP.^7^

### Data collection

Clinical characteristics, including GA, birth weight, Apgar scores at 1 min and 5 min, sex, delivery mode, small-for-GA (SGA; birth weight below the 10th percentile), pregnancy-induced hypertension, gestational diabetes mellitus and antenatal steroid use, were analyzed. GA was determined based on the mother’s last menstrual period and the result of the modified Ballard test. We investigated short-term outcomes, including death before discharge, bronchopulmonary dysplasia (≥ moderate)^27^, intraventricular hemorrhage (IVH) (≥ grade 3)^28^, necrotizing enterocolitis (≥ Bell’s stage 3b)^29^, and retinopathy of prematurity (ROP)^30^ that requires laser treatment.

The head circumference, height, and body weight of the infants at a CA of two years were measured for growth assessment. These values were converted to sex- and age-specific z scores of weight, height, and head circumference using the lambda, mu, and sigma method and the 2007 Korean National Growth Charts database^31^. Catch-up growth was defined as weight, height, or head circumference that exceeded the 10th percentile, according to the 2007 Korean National Growth Charts. Neurodevelopmental outcomes, including hearing loss that requires aids, use of eyeglasses, strabismus, Bayley scores (Bayley Scales of Infant Development, Second Edition, BSID-II), and cerebral palsy, were assessed as well. Cerebral palsy was defined as a Palisano gross motor function score ≥ 2. We classified neurodevelopmental impairment (NDI) into three groups: none or mild, moderate, and severe^32^. None or mild NDI was defined as BSID-II mental developmental index (MDI) and psychomotor developmental index (PDI) scores of 85 or greater without hearing impairment, visual impairment, and cerebral palsy. Moderate NDI was defined as mild or moderate cerebral palsy of Gross Motor Function Classification System level 2–3, or MDI or PDI scores of 70–84. Severe NDI was defined as blindness or deafness or severe cerebral palsy of Gross Motor Function Classification System level 4 or 5, or MDI or PDI scores less than 70. We reviewed chart and follow-up data to determine the NDI classifications for infants with missing or incomplete data.

Regarding TFTs, we defined THOP as a temporarily low initial T4 level < 4.5 ng/dl and/or a free T4 (fT4) level < 0.9 ng/dl with a TSH level < 20.0 µIU/ml. Severe THOP was defined as an initial T4 level < 2.5 ng/dl and/or a fT4 level < 0.5 ng/dl^7,33^. TFT normalization was defined as 0.7–7.0 μIU/ml for TSH level, 4.5–12.5 ng/dl for T4 level, and 0.9–1.8 ng/dl for fT4 level. Subsequent TFTs were performed every 2–6 weeks until hospital discharge. Levothyroxine replacement therapy for THOP was initiated at a dosage of 10–15 μg/kg/day, based on recommendations from the attending pediatric endocrinologists.

### Statistical analysis

Continuous variables were compared using Student’s *t*-test or the Mann–Whitney U test and are presented as means ± standard deviation. Categorical variables were compared using the chi-square or Fisher’s exact test and are presented as percentages and frequencies. SPSS version 25 (SPSS Inc., Chicago, IL, USA) was used for all statistical analyses. *P* < 0.05 was considered statistically significant.

## Results

### Clinical characteristics of the infants

Table 1 shows the demographic and clinical data of the infants with THOP categorized into groups of those who received levothyroxine treatment and those who did not. The mean GA and birth weight of total enrolled infants were 25.0 ± 1.4 weeks and 735.1 ± 150.0 g, respectively. Those in the treated group had significantly lower GA and birth weight, significantly more males, and a higher incidence of pathologic chorioamnionitis than those in the non-treated group. Group 1 (more severe THOP; T4 < 2.5 ng/dl and/or fT4 < 0.5 ng/dl) had significantly lower GA, birth weight, 5 min Apgar score, and incidence of antenatal steroid use; significantly more males; and a higher number of SGA infants than did group 2 (less severe THOP group) (2.5 ng/dl ≤ T4 < 4.5 ng/dl and/or 0.5 ng/dl ≤ fT4 < 0.9 ng/dl), regardless of whether they received levothyroxine replacement therapy.

**Table 1:** Demographic characteristics of the infants with transient hypothyroxinemia of prematurity.

Variable	T4 < 2.5 ng/dl and/or f T4 < 0.5 ng/dl (Group 1)			2.5 ≤ T4 < 4.5 ng/dl and/or 0.5 ≤ fT4 < 0.9 ng/dl (Group 2)			Total	
	Non-treated (N = 96)	Treated (N = 46)	Total (N = 142)	Non-treated (N = 166)	Treated (N = 27)	Total (N = 193)	Non-treated (N = 262)	Treated (N = 73)
GA (weeks)	24.6 ± 1.3	24.2 ± 1.1	**24.4 ± 1.3**	**25.5 ± 1.3**	**24.7 ± 1.2**^**a**^	**25.4 ± 1.3**^**b**^	**25.1 ± 1.4**	**24.4 ± 1.2**^**a**^
BW (g)	681 ± 143	658 ± 138	**674 ± 142**	784 ± 141	757 ± 135	**781 ± 140**^**b**^	**746 ± 150**	**695 ± 144**^**a**^
Male	53 (55)	30 (65)	**83 (59)**	76 (46)	16 (59.3)	**92 (47)**^**b**^	**129 (49)**	**46 (63)**^**a**^
1-min AS	**3.7 ± 1**	**4.2 ± 1.5**^**b**^	**3.9 ± 1.4**	4.2 ± 1.8	4.7 ± 1.4	**4.3 ± 1.8**^**b**^	4.0 ± 1.7	4.4 ± 1.5
5-min AS	6.5 ± 1.5	6.7 ± 1.4	**6.6 ± 1.5**	6.9 ± 1.5	7.0 ± 1.5	**6.9 ± 1.5**^**b**^	6.7 ± 1.5	6.8 ± 1.4
C/sec	80 (83)	38 (83)	118 (83)	126 (76)	18 (66.7)	145 (75)	206 (79)	56 (77)
SGA	20 (21)	4 (9)	**24 (17)**	16 (10)	0 (0)	**16 (8.2)**^**b**^	36 (14)	4 (6)
Antenatal steroids	70 (73)	34 (74)	**104 (73)**	137 (83)	23 (85.2)	**160 (83)**^**b**^	207 (79)	57 (78)
Pathologic chorioamnionitis	39 (41)	25 (54)	64 (45)	71 (43)	17 (63.0)	88 (45)	110 (42)	42 (58)
PIH	11 (12)	3 (7)	35 (13)	23 (14)	2 (7.4)	5 (7)	34 (13)	5 (7)
GDM	2 (2)	4 (9)	6 (4)	4 (2)	0 (0)	4 (2)	6 (2)	4 (6)

Values are presented as means ± SD or n (%).

Significant values are in [bold].

*T4* thyroxine, *fT4* free thyroxine, *GA* gestational age, *BW* birth weight, *AS* Apgar score, *SGA* small for gestational age, *PIH* pregnancy-induced hypertension, *GDM* Gestational diabetes mellitus.

^a^*p* < 0.05 compared with non-tereated group.

^b^*p* < 0.05 compared with group 1.

### Thyroid function test results of the infants

Table 2 shows the alterations in T4, fT4, and TSH levels of infants with THOP who received levothyroxine treatment and those who did not. Significantly lower initial T4 and TSH levels were observed in the treated group than in the non-treated group. Days of normalized TFT were significantly shortened in treated infants compared with non-treated infants in group 1 but not in group 2.

**Table 2:** Thyroid function test data of infants with transient hypothyroxinemia of prematurity.

Variable	T4 < 2.5 ng/dl and/or f T4 < 0.5 ng/dl (Group 1)			2.5 ≤ T4 < 4.5 ng/dl and/or 0.5 ≤ fT4 < 0.9 ng/dl (Group 2)			Total	
	Non-treated (N = 96)	Treated (N = 46)	Total (N = 142)	Non-treated (N = 166)	Treated (N = 27)	Total (N = 193)	Non-treated (N = 262)	Treated (N = 73)
**Initial TFT**								
Age, HD	7.8 ± 2.1	7.7 ± 3.5	7.7 ± 2.6	7.6 ± 3.1	7.2 ± 2.8	7.5 ± 3.0	7.7 ± 2.8	7.5 ± 3.2
T4	1.6 ± 0.6	1.6 ± 0.6	**1.6 ± 0.6**	**3.4 ± 0.6**	**3.1 ± 0.4**^**a**^	**3.4 ± 0.6**^**b**^	**2.6 ± 1.1**	**2.1 ± 0.9**^**a**^
fT4	0.3 ± 0.1	0.3 ± 0.1	**0.3 ± 0.1**	0.6 ± 0.1	0.6 ± 0.1	**0.6 ± 0.1**^**b**^	0.6 ± 0.1	0.4 ± 0.2
TSH	1.7 ± 1.7	1.3 ± 1.2	**1.6 ± 2.7**	**2.8 ± 3.1**	**1.8 ± 1.7**^**a**^	**2.7 ± 3.0**^**b**^	**2.4 ± 2.7**	**1.5 ± 1.4**^**a**^
**TFT at the start of medication**								
Age, HD	–	19.0 ± 7.4	–	–	26.4 ± 21.8	–	–	21.2 ± 14.8
T4		**3.5 ± 3.4**			**8.2 ± 0.2**^**b**^			4.6 ± 3.7
fT4		**0.8 ± 0.4**			**1.3 ± 0.4**^**b**^		–	1.0 ± 0.5
TSH		**5.3 ± 5.0**			**2.3 ± 1.3**^**b**^		–	4.2 ± 4.3
Duration of medication	–	329 ± 302	–	–	347 ± 294	–	–	336 ± 297
Normalized TFT, HD	**42 ± 31**	**33 ± 10**^**a**^	39 ± 26	49 ± 57	52 ± 71	50 ± 59	46 ± 48	40 ± 45

Values are presented as means ± SD.

Significant values are in [bold].

*T4* thyroxine, *fT4* free thyroxine, *TFT* thyroid function test, *HD* hospital day, *TSH* thyroid-stimulating hormone.

^a^*p* < 0.05 compared with non-tereated group.

^b^*p* < 0.05 compared with group 1. T4 and fT4 as µg/dl, TSH as µIU/ml.

### Short-term outcomes

Table 3 shows the clinical outcomes of THOP in infants who received levothyroxine treatment and in those who did not. Mortality and composite morbidities, including IVH and ROP, were significantly higher in group 1 than in group 2 regardless of whether the infants received levothyroxine treatment. However, mortality and composite morbidities were not higher in the treated group than in the non-treated group.

**Table 3:** Short-term outcomes.

Outcome	T4 < 2.5 ng/dl and/or f T4 < 0.5 ng/dl (Group 1)			2.5 ≤ T4 < 4.5 ng/dl and/or 0.5 ≤ fT4 < 0.9 ng/dl (Group 2)			Total	
	Non-treated (N = 96)	Treated (N = 46)	Total (N = 142)	Non-treated (N = 166)	Treated (N = 27)	Total (N = 193)	Non-treated (N = 262)	Treated (N = 73)
Mortality	26 (27)	10 (22)	**36 (25)**	11 (7)	3 (11)	**14 (7)**^**b**^	37 (14)	13 (18)
BPD (≥ moderate)	52 (54)	27 (59)	79 (56)	85 (51)	16 (59)	101 (52)	137 (52)	43 (59)
IVH (≥ grade 3)	24 (25)	13 (28)	**37 (26)**	24 (15)	7 (26)	**31 (16)**^**b**^	48 (18)	20 (27)
PVL	15 (16)	8 (17)	23 (16)	18 (11)	6 (22)	24 (12)	33 (13)	14 (19)
NEC (≥ stage 3b)	5 (5)	4 (9)	9 (6)	7 (4)	2 (7)	9 (5)	12 (5)	6 (8)
ROP that requires laser treatment	42/88 (48)	19/40 (48)	**61/128 (48)**	53/165 (32)	10/26 (39)	**63/191 (33)**^**b**^	95/253 (38)	29/66 (44)
Composite morbidities	77 (80)	34 (74)	**111 (78)**	120 (72)	21 (78)	**141 (73)**^**b**^	197 (75)	55 (85)

Values are presented as n (%).

Composite morbidities = BPD (≥ moderate) + IVH (Gr ≥ 3) + NEC (≥ 3b) + ROP that requires laser therapy.

Significant values are in [bold].

*T4* thyroxine, *fT4* free thyroxine, *BPD* bronchopulmonary dysplasia, *IVH* intraventricular hemorrhage, *PVL* periventricular leukomalacia, *NEC* necrotizing enterocolitis, *ROP* retinopathy of prematurity.

^a^*p* < 0.05 compared with non-tereated group.

^b^*p* < 0.05 compared with group 1.

### Long-term outcomes

Table 4 shows the long-term growth and neurodevelopmental outcomes of THOP in the available 208 out of 335 infants at a CA of 2 years. Non-treated infants showed significantly higher catch-up growth rate in weight and height than treated infants in group 2.

**Table 4:** Growth and neurodevelopmental outcomes at a corrected age of two years.

	T4 < 2.5 ng/dl and/or f T4 < 0.5 ng/dl (Group 1)			2.5 ≤ T4 < 4.5 ng/dl and/or 0.5 ≤ fT4 < 0.9 ng/dl (Group 2)			Total	
	Non-treated (N = 52)	Treated (N = 34)	Total (N = 86)	Non-treated (N = 100)	Treated (N = 22)	Total (N = 122)	Non-treated (N = 152)	Treated (N = 56)
WT at birth (g)	690 ± 135	659 ± 143	**678 ± 138**	777 ± 135	756 ± 137	**773 ± 135**^**b**^	**747 ± 141**	**697 ± 147**^**a**^
WT at CA 2 years (Z-score)	− 1.1 ± 1.3	− 1.6 ± 1.8	− 1.3 ± 1.5	− 1.2 ± 1.2	− 1.4 ± 1.2	− 1.2 ± 1.2	− 1.1 ± 1.2	− 1.5 ± 1.6
WT catch-up at CA 2 years	27 (52)	15 (44)	42 (49)	**56 (56)**	**8 (36)**^**a**^	64 (52)	83 (55)	23 (41)
HT at birth (cm)	31.8 ± 2.4	31.7 ± 2.0	**31.7 ± 2.3**	33.2 ± 2.3	33.0 ± 1.9	**33.2 ± 2.2**^**b**^	32.7 ± 2.4	32.2 ± 2.1
HT at CA 2 years (Z-score)	**− 0.6 ± 1.2**	**− 1.2 ± 1.5**^**a**^	− 0.8 ± 1.4	− 0.5 ± 0.9	− 0.9 ± 1.0	− 0.6 ± 1.0	**− 0.5 ± 1.0**	**− 1.1 ± 1.3**^**a**^
HT catch-up at CA 2 years	**34/48 (71)**	**15 (44)**^**a**^	**49/82 (60)**	**79/99 (80)**	**11/21 (52)**^**a**^	**90/120 (74)**^**b**^	**113/148 (76)**	**26/55 (47)**^**a**^
HC at birth (cm)	22.5 ± 2.4	22.0 ± 2.2	**22.3 ± 2.3**	23.0 ± 1.7	22.3 ± 1.9	**22.9 ± 1.7 **^**b**^	22.8 ± 2.0	22.1 ± 2.1
HC at CA 2 years (Z-score)	**− 0.9 ± 1.2**	**− 1.6 ± 1.2**^**a**^	− 1.2 ± 1.2	− 0.8 ± 1.1	− 1.4 ± 2.0	− 0.9 ± 1.3	**− 0.9 ± 1.1**	**− 1.5 ± 1.5**^**a**^
HC catch-up at CA 2 years	23 (44)	14 (41)	37 (43)	49 (49)	6 (27)	55 (44.7)	72 (47)	20 (36)
Hearing loss	0 (0)	1 (2)	1 (1)	0 (0)	0 (0)	0 (0)	0 (0)	1 (2)
Use of eyeglasses	**10 (19)**	**15 (44)**^**a**^	**25 (29)**	**7 (7)**	**6 (27)**^**a**^	**13 (11)**^**b**^	17 (11)	21 (38)
Strabismus	12 (23)	5 (15)	17 (20)	21 (21)	5 (23)	26 (21)	33 (21.7)	10 (18)
Blindness	0 (0)	0 (0)	0 (0)	0 (0)	0 (0)	0 (0)	0 (0)	0 (0)
**Cerebral palsy**	8 (15)	4 (12)	12 (14)	8 (8)	2 (9)	10 (8)	16 (11)	6 (11)
GMFCS 0–1	44 (85)	30 (88)	74 (86)	92 (92)	20 (91)	112 (92)	136 (90)	50 (89)
GMFCS 2–3	7 (14)	2 (6)	9 (11)	6 (6)	1 (4.5)	7 (6)	13 (9)	3 (5)
GMFCS 4–5	1 (2)	2 (6)	3 (4)	2 (2)	1 (4.5)	3 (3)	3 (2)	3 (5)
**Neurodevelop-mental impairment**								
No or mild	30 (58)	19 (56)	49 (57)	65 (65)	10 (46)	75 (62)	95 (63)	29 (52)
Moderate	11 (21)	3 (9)	14 (16)	19 (19)	6 (27)	25 (21)	30 (20)	9 (16)
Severe	11 (21)	12 (35)	23 (27)	16 (16)	6 (27)	22 (18)	27 (18)^a^	18 (32)

Values are presented as means ± SD or n (%).

Significant values are in [bold].

*T4* thyroxine, *fT4* free thyroxine, *WT* weight, *CA* corrected age, *HT* height, *HC* head circumference, *MDI* mental developmental index, *PDI* psychomotor developmental index.

^a^*p* < 0.05 compared with non-tereated group.

^b^*p* < 0.05 compared with group 1.

In group 1 analysis, non-treated infants showed significantly higher catch-up growth rate in head circumference than treated infants. Regarding neurodevelopmental outcomes, there were no significant differences in the incidence of cerebral palsy and neurodevelopmental delays, as evidenced by MDI and/or PDI < 70, between the non-treated and treated infants in group 1 and group 2.

## Discussion

We conducted this study to determine whether levothyroxine replacement therapy is associated with beneficial short- and/or long-term outcomes in ELBW infants with THOP. The results of this study showed that while levothyroxine replacement therapy did not improve short- and/or long-term adverse outcomes in ELBW infants with THOP, more severe THOP was associated with significantly higher acute mortality and composite morbidities, including IVH and ROP, and failure to achieve catch-up height at a CA of two years than less severe THOP, regardless of whether the infant received levothyroxine treatment. Previous research has shown that levothyroxine supplementation does not attenuate acute mortality or improve long-term neurodevelopmental outcomes, a finding which is in accordance with our results^22,34^. Taken together, these findings suggest that the severity of THOP itself rather than levothyroxine replacement therapy may be the indicator for adverse short- and/or long-term outcomes in ELBW infants with THOP.

The indications, timing, and dose of levothyroxine treatment could be the confounding variables that affect the outcomes of THOP. In the present study, levothyroxine replacement therapy was arbitrarily determined by the attending endocrinologist without any clear treatment criteria. However, the much smaller number of treated infants with less severe THOP (27/193 [14%]) than the number of treated infants with more severe THOP (46/142 [32%]) suggests that the indications for levothyroxine treatment were determined according to the severity of THOP and were subject to selection bias, thereby contributing to the lack of levothyroxine treatment benefits observed in this study. Nonetheless, this study showed that levothyroxine treatment does not attenuate adverse outcomes, even in analysis of infants with more severe THOP. Regarding timing, Iijima recommended levothyroxine treatment only if THOP is associated with elevated TSH levels^35^. However, levothyroxine treatment for THOP, which was initiated at an average of postnatal days 21 and 36 before and after elevation of TSH level, respectively, in the present study and in previous studies^33^, did not attenuate adverse outcomes. Regarding dose, 10–15 ug/kg/day, an optimal dose recommended for congenital hypothyroidism, was used in the present study^35,36^. However, the significantly shorter time intervals for normalizing TFTs in the more severe THOP group than that in the less severe THOP group may have attributed to the longer follow-up TFT intervals in the less severe THOP group. Further adequately-powered, prospective, randomized, and placebo-controlled trials are necessary to resolve these clinical paradoxes.

In previous studies, the prevalence of adverse outcomes was dependent on initial (f) T4 levels, with patients with the lowest (f) T4 levels showing the highest incidences of mortality and morbidity^33,37^, and worse neurodevelopmental outcomes until the age of 5 years^38^. In the present study, the significantly lower (f) T4 levels, GA, birth weight, and incidence of antenatal steroid use, the higher number of males, and the higher number of SGA infants in the more severe THOP group resulted in significantly increased incidences of acute mortality and morbidities, including IVH and ROP, in the more severe THOP group compared with the less severe THOP group, regardless of whether the infants received levothyroxine treatment. These findings suggest that (f) T4 levels could reflect the severity of the non-thyroidal illness in ELBW infants^7,10,33,37–43^. Moreover, as noted in other previous studies, we previously observed significantly reduced prevalence of THOP over time and better clinical outcomes as a result of continuing improvements in perinatal and neonatal intensive care medicine, including increased antenatal steroid use, better delivery room resuscitation, and application of a less invasive ventilator management policy for ELBW infants^7,26,33,44–46^. Overall, these findings suggest that THOP may simply represent an epiphenomenon of non-thyroidal illness rather than cause short- and/or long-term adverse outcomes in ELBW infants. Thus, the prevalence of THOP and its ensuing adverse outcomes could be reduced by attenuating non-thyroidal illness with better clinical management rather than administering levothyroxine treatment to ELBW infants. Currently, there is an ongoing debate whether levothyroxine replacement therapy is necessary to improve the outcomes of the non-thyroidal illness not only in the preterm infants ^20–23^ but also in the adult patients^47^. Further well- designed prospective clinical trials will be necessary to clarify this.

Measurements of T4 and fT4 levels could be confounded by the alterations in binding proteins including thyroid binging globulin^48^, and albumin^49^, and medications such as heparin and corticosteroids^7,33,50^, and the critical conditions such as COVID-19 causing non-thyroidal illness^50^. In this study, we have observed 20 cases (23%) of low T4 levels along with normal fT4 levels out of 88 simultaneous T4 and fT4 measurements. Further studies would be necessary whether this discrepancy could be explained by medications, major alterations in circulating proteins including albumin and the non-thyroidal illness itself.

This study had several limitations. First, this study was limited by its single-center, retrospective, and uncontrolled observational design. Second, there were no clear indications for levothyroxine replacement therapy, which depended entirely on the clinical decision of the attending endocrinologist. Third, the bias in the selection of the small number of treated infants in the less severe THOP group may have contributed to the results showing no benefit of levothyroxine treatment. Furthermore, wide and heterogeneous follow-up TFT intervals (every 2–6 weeks), especially in the less severe THOP group, may not have allowed for accurate comparison of the TFT normalization timing between the study groups. Nonetheless, the relatively large sample of 335 ELBW infants born with THOP and the same clinical management policy used to evaluate the benefits of levothyroxine treatment for THOP may be strengths of this study.

## Conclusion

This study showed that levothyroxine replacement therapy did not improve short- or long-term outcomes in ELBW infants with THOP. In addition, the results showed that the outcomes of THOP are dependent on the severity of the disease, in infants with more severe THOP showing significantly more adverse short-term outcomes, such as mortality and composite morbidities, and long-term outcomes, such as failure to catch-up growth for height at a CA of 2 years, than those with less severe THOP, whether they received levothyroxine treatment or not. However, considering the limitations of this study including the retrospective observational design with no clear indications for levothyroxine replacement therapy, further well-designed prospective controlled studies would be necessary to clarify the benefits of levothyroxine replacement regardless of the severity of THOP.

## Data Availability

The data that support the findings of this study are available from the corresponding author (wspark6@gmail.com) upon reasonable request.
